# Epigenetics meets GPCR: inhibition of histone H3 methyltransferase (G9a) and histamine H_3_ receptor for Prader–Willi Syndrome

**DOI:** 10.1038/s41598-020-70523-y

**Published:** 2020-08-11

**Authors:** David Reiner, Ludwig Seifert, Caroline Deck, Roland Schüle, Manfred Jung, Holger Stark

**Affiliations:** 1 Institute of Pharmaceutical and Medicinal Chemistry, Heinrich Heine University Duesseldorf, Universitaetsstr. 1, 40225 Duesseldorf, Germany; 2 Institute of Pharmaceutical Sciences, University of Freiburg, 79104 Freiburg, Germany; 3 Department of Urology, Center for Clinical Research, Medical Center, Signalling Research Centres BIOSS and CIBSS, University of Freiburg, 79106 Freiburg, Germany

**Keywords:** Drug screening, Medicinal chemistry, Pharmaceutics, Small molecules, DNA methylation, Molecular neuroscience, Growth disorders, Metabolic syndrome, Drug development, Chemical tools, Receptor pharmacology, Mechanism of action, Small molecules, Drug discovery and development, Pharmacology, Structure-based drug design, Target identification

## Abstract

The role of epigenetic regulation is in large parts connected to cancer, but additionally, its therapeutic claim in neurological disorders has emerged. Inhibition of histone H3 lysine *N*-methyltransferase, especially G9a, has been recently shown to restore candidate genes from silenced parental chromosomes in the imprinting disorder Prader–Willi syndrome (PWS). In addition to this epigenetic approach, pitolisant as G-protein coupled histamine H_3_ receptor (H_3_R) antagonist has demonstrated promising therapeutic effects for Prader–Willi syndrome. To combine these pioneering principles of drug action, we aimed to identify compounds that combine both activities, guided by the pharmacophore blueprint for both targets. However, pitolisant as selective H_3_R inverse agonist with FDA and EMA-approval did not show the required inhibition at G9a. Pharmacological characterization of the prominent G9a inhibitor A-366, that is as well an inhibitor of the epigenetic reader protein Spindlin1, revealed its high affinity at H_3_R while showing subtype selectivity among subsets of the histaminergic and dopaminergic receptor families. This work moves prominent G9a ligands forward as pharmacological tools to prove for a potentially combined, symptomatic and causal, therapy in PWS by bridging the gap between drug development for G-protein coupled receptors and G9a as an epigenetic effector in a multi-targeting approach.

## Introduction

Prader–Willi syndrome (PWS) is a rare neurogenetic disorder that affects approximately 1 of 15,000–30,000 newborn infants^1,2^. Clinically, the disease manifests in a marked hypotonia that presents as earliest symptoms in reduced fetal movement, in sucking weakness of neonates and further limits motoric development in early childhood^3^. Following a period of reduced nutrition due to decreased muscle tone, the disease proceeds with a bland feeling of satiety, leading to a massive urge for eating (hyperphagia). If not controlled by exogenic dietary limitation through caregivers, PWS leads to obesity during adolescence and adulthood, that is the common reason for increased morbidity and mortality of such patients^1,3^. Next to behavioral disorders, patients often show mild mental retardation such as restraints in executing complex tasks and/or mildly reduced intelligence, short stature, hypogonadism, a general delayed development and sleeping issues that demonstrate as hypersomnia and excessive daytime sleepiness (EDS)^3–5^.

PWS is referred to as a neurogenetic disorder that has been associated with a loss of genetic information between loci q11 and q13 on the chromosome 15 where several SNORD clusters and the genes SNURF-SNRPN, NDN, MKRN3 and MAGEL2 are located^6^. However, it is hard to correlate their loss with specific symptoms of the phenotype. On the one hand, their specific functions have not been elucidated yet, on the other hand, not many PWS or PWS-like phenotypes could be attributed to the loss of a single of such genes^7^. The function of the small nucleolar RNA (snoRNA) expressed by SNORD116 has not been elucidated yet, though, the deletion of this cluster suggests a critical role for determining the PWS phenotype^8,9^. Progress of knowledge about the organization of genes led to an understanding of the molecular origin of PWS. It is caused by a loss of the paternally inherited genes within the depicted loci, either due to deletion or uniparental disomy. At the same time, the copy of information remains on the corresponding maternal chromosome^10^. However, the genes on this opposite parental chromosome are silenced by epigenetic mechanisms, such as DNA methylation or post-translational histone modifications that lead to imprinting of the corresponding alleles. Thus, PWS is referred to as an “imprinting disorder”, a group of disorders that shares many clinical manifestations such as affected growth, development, metabolism or behavior^11^.

The current pharmacotherapeutic interventions in PWS involve substitution of Growth Hormone that has shown to improve body composition and motoric strength. It can and should be applied before the first birthday of infants^1^. Additionally, the application of sexual hormones, antipsychotics and antidepressants in the disease is reported in the literature^1^. Among the psychiatric drugs, modafinil demonstrated effectiveness to relieve the impulsive behavior of PWS patients and has been approved for the treatment of EDS or narcolepsy^12^. Similarly, application of pitolisant as novel inverse agonist/antagonist at the G-protein coupled histamine H_3_ receptor (H_3_R) by children suffering from PWS is known to us^13–15^. The drug obtained market-approval by the European Medicines Agency of the European Union (EMA) in 2016 for narcolepsy with or without cataplexy, recently followed by the FDA (U.S. Food and Drug Administration) approval. In clinical studies, the drug displayed significant improvement of EDS determined by the Epworth Scale of Sleepiness (ESS) and non-inferiority towards the therapeutically established modafinil^16^. Additionally, pitolisant is currently examined for effects in pediatric narcoleptic patients (ClinicalTrials.gov database of the U.S. National Institutes of Health, Identifier: NCT02611687, https://www.clinicaltrials.gov/). Though highly significant clinical studies for pitolisant in PWS patients are missing to date, recent patient-based case reports suggest benefits of this H_3_R targeting drug. It shows improved activity of patients, reduction of daytime-sleepiness as well as improvements in mental clarity and processing speed^13–15^. Moreover, preclinical in vivo examination in SNORD116-deficient PWS mice showed abolished baseline changes in REM sleep after administration of pitolisant^17^ that has emphasized the role of H_3_R in the pathophysiology of PWS.

The outlined therapeutic options are mainly linked to decrease behavioral and endocrinal symptoms; however, without clear evidence for each of them. Therefore, appropriate and causal pharmacotherapy for PWS is still demanded.

On this search, the demonstration of an epigenetic approach to PWS by Kim and co-workers in 2017 has got our attention^10,18^. The group shows that at least two inhibitors of the histone H3 lysine-9 (H3K9) methyltransferase G9a (syn. Euchromatic histone *N*-methyltransferase 2, EHMT-2) are capable of restoring the expression of candidate PWS genes from the maternally inherited chromosome. While the group found no alterations in the level of DNA-methylation within the imprinted region and genes were still restored, the role of methylation for gene silencing seems less important in PWS. Therefore, the relevance of histone H3 methylation as a regulator of the expression of the imprinted genes during imprinting has been highlighted^18^. Further unknown roles of G9a to gene expression may contribute^19^, and involvement of additional regulators of gene expression seems likely. For example, some G9a inhibitors have shown inhibition of Spindlin1 that belongs to the epigenetic “reader” proteins and has been studied for its role in cancer progression^20^. It can detect H3K4 trimethylation (H3K4me3) and trigger downstream signalling^21^ as well as the expression of rRNA genes^22^.

Inspired by the recent progress of pitolisant in PWS, we aimed to accelerate the preclinical and clinical investigation by the discovery of further H_3_R inverse agonists/antagonists with improved profiles. The reported potential of the G9a inhibitors UNC-0642 and UNC-0638 to restore the expression of candidate genes in PWS prompted us to identify H_3_R antagonists among compounds with inhibitory activity for G9a. Additionally, we took identified lead-compounds for a selectivity screening among histamine H_4_ receptors that possess high structural similarity to H_3_R^23^ as well as towards dopaminergic receptor subtypes that have been associated with the regulation of food intake^24^. Finally, relevant queries were made for Spindlin1 inhibition to identify congeners for further pharmacological elucidation of involvement of this target in PWS.

## Results

### Cross-over screening of H_3_R ligands and dual G9a/Spindlin1 inhibitors

Testing of the H_3_R ligands pitolisant and ciproxifan did not reveal remarkable inhibition of G9a and Spindlin1. UCL-2190 showed only slight G9a inhibition when compared to negative control (buffer only, *P* = 0.035). In contrast, known G9a inhibitors potently diminished H3K9 dimethylation (Table 1). Additionally, UNC-0642 inhibited Spindlin1 to interact with trimethylated H3K4 at 10 µM. Interestingly, such dual G9a/Spindlin1 inhibitors were as well able for potent displacement of [^3^H]*N*^α^-methylhistamine from human isoform of H_3_R (hH_3_R) in the nanomolar concentration range. While A-366 exerted its action at a concentration similar to the prominent H_3_R inverse agonist/antagonist pitolisant, UNC-0642 was more active by about an order of magnitude.

**Table 1:** Representative ligands and their G9a inhibition, Spindlin1 inhibition and H_3_R affinity.

	G9a methyltransferase inhibition at 10 µM^a^ (n)	Spindlin1 inhibition^b^	hH_3_R affinity *K*_i_ [CI_95%_]^c^ (n)
Ciproxifan	− 1.9 ± 4.9% (2)	No inhibition	320 [250–430] nM^47^ (3)
UCL-2190	13.9 ± 9.3% (2)	No inhibition	11 [3.5–33] nM^48^ (3)
Pitolisant	− 2.6 ± 12.2% (4)	No inhibition	12 [11–13] nM^47^ (5)
A-366	100.00 ± 0.04% (6) *IC*_50_ = 2.5 nM^28^	*IC*_50_ = 182.6 nM^37^	17 [8–37] nM (6)
UNC-0642	99.90 ± 0.09% (4)	*IC*_50_ = 2.7 ± 6.7 µM	1.8 [0.6–5.5] nM (4)

^a^AlphaLISA based CLOT (Chemiluminescence-based oxygen tunnelling) assay; results are expressed as means ± s.d. from the indicated number of replicates (n).

^b^Screening for inhibition of the epigenetic reader protein Spindlin1 in a fluorescence polarization-based approach.

^c^Affinity to the human isoform of histamine H3 receptor (hH_3_R) as determined by [^3^H]*N*^α^-methylhistamine displacement studies.

### Selectivity screening of G9a inhibitors at other GPCR subtypes

**Figure 1:**
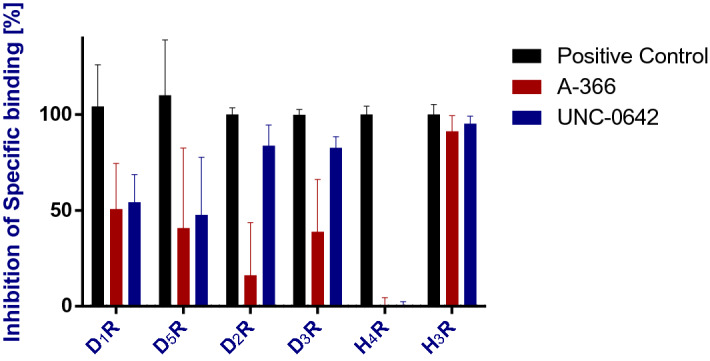
Screening for selectivity of G9a-inhibitors at 1 µM among dopamine D_1_, D_5_, D_2_ and D_3_ receptors (D_1_R, D_5_R, D_2_R, D_3_R, respectively) and at the histamine H_4_ receptor (H_4_R). For comparison, the figure depicts the inhibition of specific binding to H_3_R that was extracted from affinity screening data. Bars represent means ± s.d. of the inhibition of radioligand binding to the respective receptor by either A-366, UNC-0642 or control compound (100 µM fluphenazine for D_1_R and D_5_R, 10 µM haloperidol for D_2_R and D_3_R, 100 µM JNJ-7777120 for H_4_R or 10 µM pitolisant for H_3_R).[^3^H]-SCH23390, [^3^H]-spiperone, [^3^H]-histamine and [^3^H]*N*^α^-methylhistamine were used as radiolabelled tracers at D_1_R/D_5_R, D_2_R/D_3_R, H_4_R and H_3_R, respectively, each at approx. 1 × *K*_D_.

The G9a inhibitors A-366 and UNC-0642 were screened for their ability to inhibit binding of radiolabeled ligands to dopamine D_1_, D_2_, D_3_, D_5_ receptors (D_1_R, D_5_R, D_2_R, D_3_R) and histamine H_4_ receptor (H_4_R) (Fig. 1). For both compounds, significant differences to the respective positive controls were observed (*P* < 0.05; positive controls: 10 µM haloperidol for dopaminergic receptor subtypes, 100 µM JNJ-7777120 for histamine H_4_ receptor, 10 µM pitolisant for H_3_R). Such differences were slight with regards to H_3_R (A-366: ∆between means = 9%, UNC-0642: ∆between means = 5%). However, differences in inhibition were significantly more pronounced when comparing their activity between H_3_R and the other GPCR subtypes (P < 0.02), suggesting lower receptor affinity for the latter. A-366 and UNC-0642 did not differ from each other for their exerted radioligand displacement at H_3_R, H_4_R, D_1_R and D_5_R (*P* > 0.22). In contrast, a higher susceptibility to displace [^3^H]-spiperone from D_2_R and D_3_R was observed for UNC-0642 than for A-366 (P < 0.01).

### Mode of antagonism of A-366 at rat isoform of H_3_R

**Figure 2:**
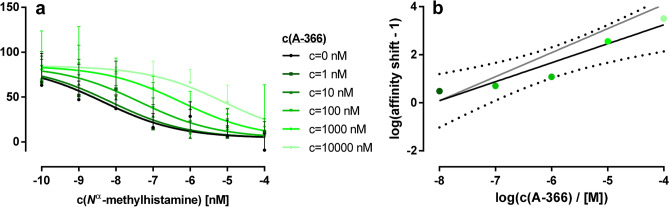
Schild-assay revealing antagonist properties of A-366 at rat isoform of H_3_R. (**a**) Effects of the H_3_R agonist *N*^α^-methylhistamine on formation of cAMP concentrations were studied in a cAMP response element-driven luciferase reporter gene (CRE-Luc) assay in HEK-293 T cells that were stably transfected with the receptor as described by Nordemann et al.^44,45^ Evaluated data originated from two independent experiments performed in duplicate and are stated as means ± s.d. (**b**) Data from panel a were transformed to a Schild-plot that resulted in a regression of R^2^ = 0.91 (black line, with 95% confidence band depicted with small dots). The slope was not different from unity (grey line).

Additionally, A-366 was investigated in a cAMP-response element driven luciferase reporter gene (CRE-Luc) assay at another isoform of H_3_R. At the *Rat Norvegicus* isoform of H_3_R (rH_3_R), A-366 potently shifted receptor activation by histamine (*EC*_50_ = 2.3 [0.4–14.9] nM). As determined from fitting data of Fig. 2a, the resulting affinity was in line with the observations above that used the human isoform of H_3_R (hH_3_R, *K*_B_ = 15 [2–150] nM). Subsequent Schild-plot showed that A-366 shifts the affinity of histamine in a rather equipotent manner (Fig. 2b) with a slightly reduced slope (0.79 ± 0.45, mean ± 95% confidence interval) but not significantly different from unity.

## Discussion

Among our search for novel H_3_R ligands with combined G9a inhibitory activity, relevant progress could be made in this study to define a novel mode of action in the pharmacotherapy of PWS. Particularly guided by the recent clinical effects of pitolisant, we started with the search for a potential epigenetic mechanism of action for pitolisant as well as ciproxifan and UCL-2190. However, such could not be delineated based on our data. Ciproxifan serves as an advanced pharmacological tool on preclinical investigation stage and a standard tool in various rodent models^25^, despite that included imidazole moiety^26^. Some drawbacks associated with the susceptibility of imidazole to inhibit CYP enzymes led to the derivative UCL-2190 that belongs to the second, nonimidazole-based generation of H_3_R antagonists^27^. Whereas a slight G9a inhibition in low percentile range was observed for UCL-2190, the corresponding affinity estimate would be far apart from such observed for potent G9a inhibitors. Crystal structures of some G9a inhibitors in complex with the enzyme suggest the necessity of protonated heterocyclic element for ionic interaction with the Asp1088 residue of the enzyme^28–30^. We attribute the lack of G9a inhibition by our scrutinized H_3_R inverse agonists to the absence of this structural feature.

In contrast, we could identify potent H_3_R ligands among G9a inhibitors. Therefore, we examined UNC-0642, bearing a quinazoline-core motif and A-366 as a spirocyclic 2-amino-3*H*-indole-based G9a-pharmacophore. The latter is suggested to be protonated at physiological pH-value due to variation towards an inherent amidine or aromatic guanidine functionality^30,31^. Further aliphatic and amino group-containing moieties are tolerated. Interestingly, for some G9a inhibitors, the core bears substituents like a 3-pyrrolidinopropoxy moiety as present in A-366 and UNC-0642. As enlightened from the crystal structures, the latter motif could be linked to an increased potency at G9a due to substrate mimicking of lysine in position 9 of histone H3K9 and therefore a blocking of the lysine binding tunnel in the histone H3 binding pocket^30^. Additionally, this variation draws the basis for an H_3_R pharmacophore that can constitute of a basic moiety, linked by an alkyl-spacer towards a substitutable aromatic central core^32^. The high binding affinity of the G9a inhibitors can be explained as such features have already been incorporated into UNC-0642 and A-366. For the former tool compound, the findings are in line with its previous characterization as a G9a inhibitor with selectivity over a broad range of kinases, transporters, ion channels as well as GPCR´s, except an affinity at histamine H_3_R^28^.

To search for potential discriminants between both pharmacological tools at GPCRs, we extended our in vitro profiling with selectivity studies against a small set of dopamine receptors (D_1_R, D_5_R, D_2_R, D_3_R) as well as the histamine H_4_ receptor that shows the highest structural similarity to H_3_R among GPCRs^23^. In all cases, inhibition of radioligand binding to the off-targets was lower for UNC-0642 and A-366 when compared with the respective positive controls at such receptors and also lower when compared to their inhibitory activity at H_3_R. In essence, one could hypothesize an additional action of agonists at D_2_R would have beneficial effects for PWS due to a suppressed food-intake in vivo^24^. Consequently, antagonists could compromise such an effect^33^. Thus, we see the selectivity against D_2_R and D_3_R, that was slightly more pronounced for A-366 than for UNC-0642, as an essential property for our desired pharmacological tools.

As a consequence of the well-documented interspecies differences of H_3_R affinity, we decided to determine A-366 binding at the rH_3_R. Due to the G_i/o_ coupling nature of H_3_R, agonists as *N*^α^-methylhistamine lead to a reduced intracellular cAMP content compared to untreated cells^34^. In a Schild-based^35^ characterization of A-366 as depicted in Fig. 2, we observed a potency that was consistent with such at hH_3_R and an equipotent affinity shift of agonist with increasing antagonist concentrations. This result creates a basis for exploitability of H_3_R mediated effects in preclinical PWS in vivo studies, although we are aware that mouse models have been predominantly used in the past. However, some reports move for extended usage of animal models other than PWS mouse models as such do usually not present obesity and hyperphagia simultaneously^36^.

Together with the previously presented data^30,37^, our results indicate that both G9a standard ligands have low nanomolar H_3_R binding affinities with required selectivity among further GPCR subtypes and that they exert potent inhibition of G9a and Spindlin1. Besides the effectiveness of UNC-0642 in PWS mice that was mentioned previously, this compound has already been subjected for further neurological examination, showing amelioration of autism-like social deficits in Shank3-deficient mice^38^ and reduction of anxiety-related behavior in adult mice^39^. In the latter study, effects similar to those of UNC-0642 could be demonstrated for A-366^39^. This implies both compounds to be tolerated in mouse or rat models and that they possess essential features for neurological drugs such as blood–brain barrier permeability and metabolic stability^18,28,30,40^. Therefore, both ligands will be suitable pharmacological tools for potential in vivo investigation.

**Figure 3:**
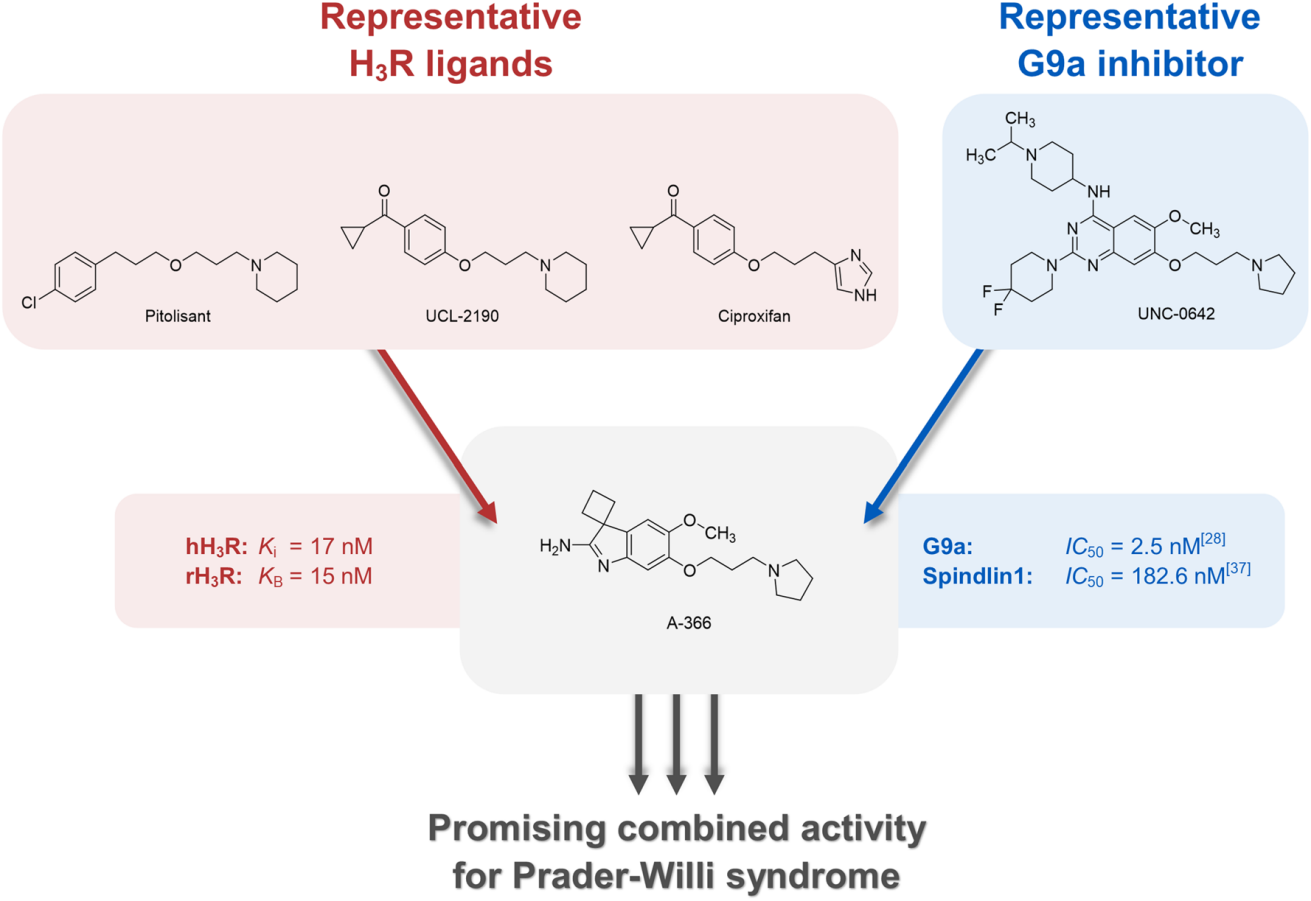
Promising combined H_3_R antagonist, G9a- and Spindlin1-inhibitor activity of A366 for pharmacotherapy of Prader–Willi syndrome (PWS).

Concluding our search for potential dual G9a inhibitors/H_3_R antagonists for the treatment of PWS in future, significant prerequisites for applying the preclinical candidate A-366 in PWS studies could be identified. With the identification of H_3_R antagonizing properties of A-366, our in vitro characterization presents this compound as a multi-target ligand that has a high potential to show symptomatic effects in the neurogenetic PWS, congruent to those described for pitolisant (Fig. 3). Secondly, the recently demonstrated gene restoration from maternal chromosomes by UNC-0642 mediated G9a inhibition should allow for a potential causal intervention by A-366. Besides, the advanced preclinical development stage of this drug makes it very attractive for further clinical characterization, promising a symptomatic and causal approach in the pharmacotherapy of PWS.

## Methods

### Materials

UCL-2190^41^, Ciproxifan^42^ and Pitolisant^27^ were from own laboratory stocks of which synthesis and analytics have been described previously. G9a-inhibitors A-366 and UNC-0642 as well as G9a enzyme, *S*-adenosylmethionine (SAM, (2*S*)-2-amino-4-[[(2*S*,3*S*,4*R*,5*R*)-5-(6-amino-9*H*-purin-9-yl)-3,4-dihydroxytetrahydrofuran-2-yl]methyl-methylsulfonio]butanoate), biotinylated histone H3 (1–21) fragment and Dulbecco´s modified eagle medium (DMEM, article no. D5671) were purchased from Sigma-Aldrich, Taufkirchen, Germany. Fetal bovine serum albumin (FBS Good-Forte) and Dulbecco´s Phosphate Buffered Saline (DPBS) were provided by PAN biotech (Aidenbach, Germany). The radioligands [^3^H]*N*^α^-methylhistamine, [^3^H]histamine, [^3^H]spiperone and [^3^H]SCH23390 were purchased from PerkinElmer (Rodgau, Germany) as well as AlphaLISA materials such as AlphaLISA H3K9me2 acceptor beads, streptavidin-coated donor beads, detection buffer (5x) and white 384-well microplates (OptiPlate). Human or animal blood/tissue/cell samples have not been used in this study.

### Cell culture and membrane preparations

Cell culture and membrane preparations for radioligand displacement assays were performed according to the protocols provided by Bautista-Aguilera et al.^43^.

HEK-293T cells were used for cAMP-response element driven luciferase reporter gene (CRE-Luc) assays, that were stably transfected with cDNA of the H_3_R isoform of *Rattus Norvegicus* (rH_3_R, NCBI sequence code: NC_005102.4) and a vector containing the *Photinus pyralis* luciferase with a cAMP-response element in its promotor region^44^. Cells were cultured in DMEM supplemented with 1% FBS in the presence of hygromycin (250 µg/mL) and geneticin (1,000 µg/mL) under culture conditions of 37.0 °C, 5.0% CO_2_-saturation and 95.0% humidity (for source of cell lines see Supplementary Information).

### Radioligand displacement assays at GPCRs

The affinity of A-366 and UNC-0642 at human isoform of H_3_R (NCBI sequence code: NM_007232.3) was determined in radioligand displacement studies at membrane preparations of transfected HEK-293 T cells. Therefore, titration schemes ranging from 0.003 to 1,000 nM were prepared in duplicates and incubated with 20 µg/200 µL protein and [^3^H]*N*^α^-methylhistamine (c = 2 nM) for 90 min. To determine non-specific binding, additional samples of pitolisant 10 µM were prepared. For off-target activity screenings, 1 µM of G9a inhibitors were incubated with receptors at the conditions that are described in Table 2. Therefore, triplicates were examined in the case of dopaminergic or histaminergic receptor subtypes, respectively.

**Table 2:** Conditions for screening of A-366 for off-target activity (dopamine D_1_, D_2_, D_3_, D_5_ and histamine H_4_ receptors).

Receptor NCBI sequence code (protein content)	Cell line	Radioligand (concentration)	Control (concentration)	Incubation time
**Dopamine D**_1_ NM_000794.5 (10 µg/200 µL)	HEK-293 T	[^3^H]SCH23390 (0.3 nM)	Fluphenazine (100 µM)	120 min
**Dopamine D**_2_ NM_016574.3 (25 µg/200 µL)	CHO-K1	[^3^H]spiperone (0.2 nM)	Haloperidol (10 µM)	120 min
**Dopamine D**_3_ NM_000796.6 (20 µg/200 µL)	CHO-K1	[^3^H]spiperone (0.2 nM)	Haloperidol (10 µM)	120 min
**Dopamine D**_5_ NM_000798.5 (5 µg/200 µL)	HEK-293 T	[^3^H]SCH23390 (0.3 nM)	Fluphenazine (100 µM)	120 min
**Histamine H**_4_ NM_021624.4 (60 µg/200 µL)	Sf9	[^3^H]histamine (10 nM)	JNJ-7777120 (100 µM)	60 min

The workflow to terminate incubation and measurement of bound radioligand was identical for both experimental set-ups. Briefly, samples were filtrated from microplates onto GF/B filters presoaked with 0.3% polyethyleneimine solution using a 96-well cell harvester. Filter mats were washed three times with water at 4 °C, dried for 60 min (54 °C), soaked with scintillation liquid (Betaplate Scint, PerkinElmer), sealed and subjected to scintillation counting.

### G9a-inhibition screening

Inhibition of G9a was examined in an AlphaLISA based format with protocols provided by PerkinElmer. In brief, compounds were incubated for 30 min on white 384-well microplates at the indicated concentration and with 5 nM G9a (Supplementary Information, Figure S1), 100 nM histone H3 (1–21) fragment and 15 µM SAM in assay buffer (50 mM Tris–HCl (pH = 9.0); 50 mM NaCl, 1 mM dithiothreitol, 0.01% Tween-20). Incubation was terminated by addition of anti-H3K9me2 acceptor beads in provided detection buffer. After incubating the mixture for 60 min, streptavidin-coated donor beads were added to the mix for additional 30 min. Luminescence was then measured using the AlphaLISA luminescence filter of an Infinite M1000pro multiplate reader (Tecan, Maennedorf, Switzerland) for 1,000 ms (integration time).

### Spindlin1 inhibition screening

Spindlin1 inhibition was determined using the fluorescence polarization displacement assay described by Wagner et al.^37^ For the *IC*_50_ values, 12 concentrations were measured in triplicates.

### CRE-Luc assays at rH_3_R

CRE-Luc assays were conducted by following the protocol provided by Nordemann et al.^44,45^, with slight modifications: For functional-based Schild^46^ studies in HEK-293T cells, such were seeded into polyethyleneimine-coated 96-well tissue culture plates (TPP) at 2 10^5^ cells/200 µL/well in assay medium (DMEM without phenol-red, 1% FBS) and allowed to attach for 24–48 h. Afterwards, forskolin (c_final_ = 3 µM) and serially-diluted *N*^α^-methylhistamine (10,000–0.01 nM) were added to the reaction cells in absence or presence of A-366 (10–100,000 nM) using a Freedom EVO^®^ liquid handling robot (Tecan). The mixture was incubated for 5 h under culture conditions.

Subsequently, the medium was removed and replaced by 80 µL lysis buffer (25 µM tricine, 10% glycerol, 2 µM egtazic acid, 1% Triton X-100, 5 µM MgSO_4_-7H_2_O and 1 µM dithiothreitol) for 30 min while shaking at 300 rpm. Lysed homogenate was transferred into white microplates. Luminescence was recorded using an Infinite M1000pro multiplate reader (Tecan) in luminescence mode (3,000 ms integration time, no filter) immediately after addition of 40 µL assay-buffer (25 mM glycylglycine, 15 mM MgSO_4_-7H_2_O, 15 mM KH_2_PO_4_, 4 mM egtazic acid, 2 mM dithiothreitol, 1 mM ATP, 50 µM coenzyme A, 0.02 mg/mL d-luciferin potassium salt) by the injector module.

### Data handling and statistics

For experiments employing radiolabeled ligands, raw data that were measured as counts-per-minute [c.p.m.] were reduced by non-specific binding. For affinity measurements, such results were fitted to least-squares method “One site competition” of GraphPad Prism version 7.0 (La Jolla, CA, United States) and final values were calculated as means [95% confidence interval]. In case of selectivity experiments, inhibition of specific binding [%] was calculated from raw data according to [1- (SM – NSB)/(TB – NSB)]*100%, where SM, NSB and TB refer to binding in the presence of ligand, non-specific binding and total binding, respectively. Data were stated as means ± s.d. For G9a inhibition studies, results were calculated from luminescence according to: 100% * [1-(SM-NC)/(PC-NC)], where SM, NC and PC refer to luminescence in samples including test compound, water and A-366 at 10 µM, respectively. Data were stated as means ± s.d. with the indicated number of replicates. For CRE-Luc assays, data were normalized to luminescence derived by forskolin containing samples (= 100%) and minimum luminescence measured in samples containing forskolin + *N*^α^-methylhistamine (10 µM) (= 0%). Data from both experiments were globally fit to the “Gaddum/Schild *EC*_50_ shift” model of GraphPad Prism and were stated as means [95% confidence interval].

Where appropriate, non-parametric tests or parametric t-tests were conducted utilizing GraphPad Prism to test for differences between data, while assuming significance if *P* < 0.05.

## Supplementary information

Supplementary file1

## Data Availability

Data for the conducted studies will be provided by the corresponding author upon reasonable request.
