# Supplementary material for: Epigenetics meets GPCR: inhibition of histone H3 methyltransferase (G9a) and histamine H3 receptor for Prader–Willi Syndrome
Source: Sci Rep. 2020 Aug 11;10:13558. doi: 10.1038/s41598-020-70523-y (PMC7419559; doi:10.1038/s41598-020-70523-y)
Supplement: Supplementary file 1 — Supplementary file1 [file 41598_2020_70523_MOESM1_ESM.pdf]

Supplementary Information

Epigenetics meets GPCR – Inhibition of histone H3 methyltransferase (G9a) and histamine H_3 receptor for Prader-Willi Syndrom

David Reiner[1], Ludwig Seifert[2], Caroline Deck[2], Roland Schüle[3], Manfred Jung[2], Holger Stark[1,*]

[1] Institute of Pharmaceutical and Medicinal Chemistry, Heinrich Heine University Duesseldorf, Universitaetsstr. 1, 40225 Duesseldorf, Germany;
[2] Institute of Pharmaceutical Sciences, University of Freiburg, 79104 Freiburg, Germany
[3] Department of Urology, Center for Clinical Research, Medical Center, Signalling Research Centres BIOSS and CIBSS, University of Freiburg, 79106 Freiburg, Germany

*Corresponding author: Holger Stark, Tel.: +49 211 81 10478, Fax: +49 211 81 13359, Email: stark@hhu.de.

AlphaLISA™ based G9a inhibition

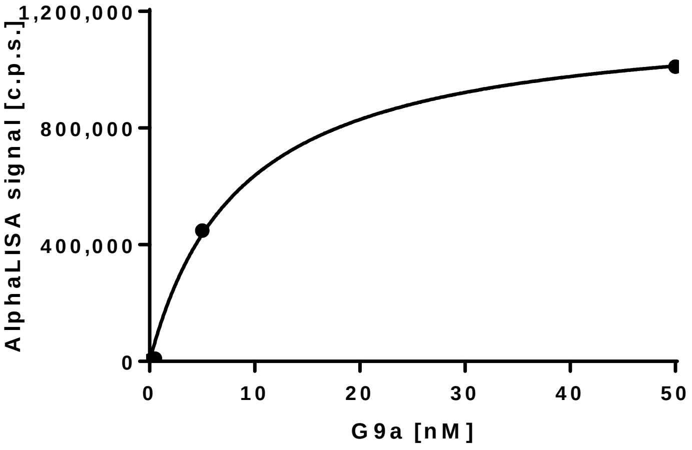

Figure S1. G9a enzyme titration (0.05, 0.5, 5 and 50 nM) at fixed concentrations of recombinant histone H3 fragment 1-21 (c = 100 nM) and *S*-adenosylmethionine (SAM, c = 15 µM). Incubation was carried out once for 30 min. Data represent means ± s.d. from an initial experiment performed in duplicates. For further tests, 5 nM G9a were used.
